# IL-17 Cytokines Induce IκBζ in Dermal Fibroblasts to Promote Pro-Inflammatory Gene Expression in Psoriasis

**DOI:** 10.3390/ijms27031297

**Published:** 2026-01-28

**Authors:** Lejla Svraka, Anna Skarnvad Andersen, Toke Touborg, Thomas Emmanuel, Udayaraja GK, Haja N. Kadarmideen, Trine Bertelsen, Christian Vestergaard, Claus Johansen

**Affiliations:** 1 Department of Dermatology, Aarhus University Hospital, 8200 Aarhus, Denmark; lejlasvraka@clin.au.dk (L.S.); annaa8@rm.dk (A.S.A.); toketouborg@clin.au.dk (T.T.); thomas.emmanuel@clin.au.dk (T.E.); bertelsen.trine@gmail.com (T.B.); christian.vestergaard@clin.au.dk (C.V.); 2 Department of Clinical Medicine, Aarhus University, 8000 Aarhus, Denmark; 3 Department of Animal and Veterinary Sciences, Aarhus University, 8830 Tjele, Denmark; udaya@anivet.au.dk (U.G.); haja.k@anivet.au.dk (H.N.K.); 4 Department of Molecular Biology and Genetics, Aarhus University, 8000 Aarhus, Denmark; 5 Hudlægecenter Nord, 9220 Aalborg, Denmark

**Keywords:** IκBζ, dermal fibroblasts, psoriasis pathogenesis, IL-17 cytokines, IL-17 signaling, TNF signaling, NF-κB pathway, fibroblast-driven inflammation, inflammation, inflammatory skin diseases

## Abstract

IκBζ (*NFKBIZ*) has been implicated as a key co-transcription factor in psoriasis pathogenesis. While its role in keratinocytes is well established, the involvement in dermal fibroblasts, another critical skin cell type, remains underexplored. This study characterizes cytokine-induced *NFKBIZ* regulation in human dermal fibroblasts in vitro and integrates spatial transcriptomics to determine *NFKBIZ* expression patterns in psoriatic skin biopsies. Primary dermal fibroblasts were stimulated with IL-17A, IL-17F, and TNF. Signaling pathways and gene regulation were examined using chemical inhibitors, siRNA knockdown, qPCR, and Western blotting. Additionally, spatial transcriptomics (CosMx™) assessed *NFKBIZ* expression in paired lesional and non-lesional psoriatic skin biopsies. Results showed significant upregulation of IκBζ expression in dermal fibroblasts following stimulation with both IL-17A and IL-17F. The NF-κB signaling pathway was identified as the primary regulator of *NFKBIZ* induction. *NFKBIZ* knockdown significantly reduced cytokine-induced expression of inflammatory mediators (CXCL8, CCL20, CCL2), confirming its regulatory role. Spatial transcriptomics further confirmed *NFKBIZ* expression in dermal fibroblasts in vivo, particularly in lesional psoriatic skin. This study establishes IκBζ as a critical modulator of inflammatory responses in dermal fibroblasts, expanding its recognized role beyond keratinocytes and immune cells, and highlights IκBζ inhibition as a potential therapeutic strategy.

## 1. Introduction

IκBζ, encoded by the *NFKBIZ* gene, is recognized as a key co-transcription factor involved in psoriasis pathogenesis [1]. In contrast to classical IκB proteins that inhibit the NF-κB pathway, IκBζ functions as a positive regulator, amplifying downstream inflammatory responses [1,2]. Elevated levels of IκBζ are consistently observed in lesional psoriatic skin, and *NFKBIZ*-/- mice exhibit resistance to experimentally induced psoriasis, underscoring its importance in disease development [1]. Moreover, the essential psoriasis-associated cytokines IL-17A and IL-17F have been demonstrated to rapidly induce the expression of *NFKBIZ* through the activation of MAPK and NF-κB signaling pathways [2], positioning IκBζ as a key downstream effector of the IL-17 axis. Consistently, the anti-psoriatic effects of IL-17A inhibition with Secukinumab have been linked to the rapid downregulation of IκBζ early after treatment initiation [3]. IκBζ mediates its regulatory function through interaction with the NF-κB subunit p50, thereby enhancing the transcriptional activity of secondary response genes through chromatin remodeling mechanisms [1]. IκBζ regulates inflammatory responses across multiple cell types. Specifically, IκBζ upregulates psoriasis-associated genes in primary human keratinocytes, including *DEFB4*, *CCL20*, *S100A7*, *IL-19*, *CXCL8*, and *LCN2* [1]. In Th17 cells, IκBζ was demonstrated to work in conjunction with RORγt to regulate the expression of IL-17A, IL-22, and TNF [4].

Despite substantial insight into the role of IκBζ in keratinocytes and Th17 cells, little is known about its function in other skin-resident cells, such as dermal fibroblasts. Fibroblasts are the predominant cell type in the dermal layers of the skin, maintaining tissue structure and function. Emerging evidence suggests that fibroblast dysregulation contributes to the pathogenesis of autoimmune skin diseases, including psoriasis [5,6]. Dermal fibroblasts have been shown to influence epidermal proliferation [7,8,9,10,11,12]. In psoriatic lesions, fibroblasts overproduce several growth factors, promoting keratinocyte hyperproliferation [7,8,9,11]. While early studies primarily focused on this hyperproliferative effect, recent advancements in single-cell RNA sequencing (scRNA-seq) have highlighted the immunomodulatory properties of fibroblasts, identifying heterogeneous subpopulations that exhibit diverse proinflammatory roles in psoriasis [13,14,15,16].

This study aims to elucidate the role of IκBζ in human dermal fibroblasts in response to psoriasis-associated cytokines and to characterize the underlying signaling pathways. We hypothesize that IκBζ is a key regulator of inflammatory gene expression in dermal fibroblasts, contributing to the amplification and persistence of psoriatic inflammation. Understanding this pathway could reveal novel mechanisms of fibroblast involvement in psoriasis and further substantiate IκBζ as a relevant therapeutic target.

## 2. Results

### 2.1. IL-17A and IL-17F Together with TNF Synergistically Induce NFKBIZ and IκBζ in Dermal Fibroblasts

**Figure 1:**
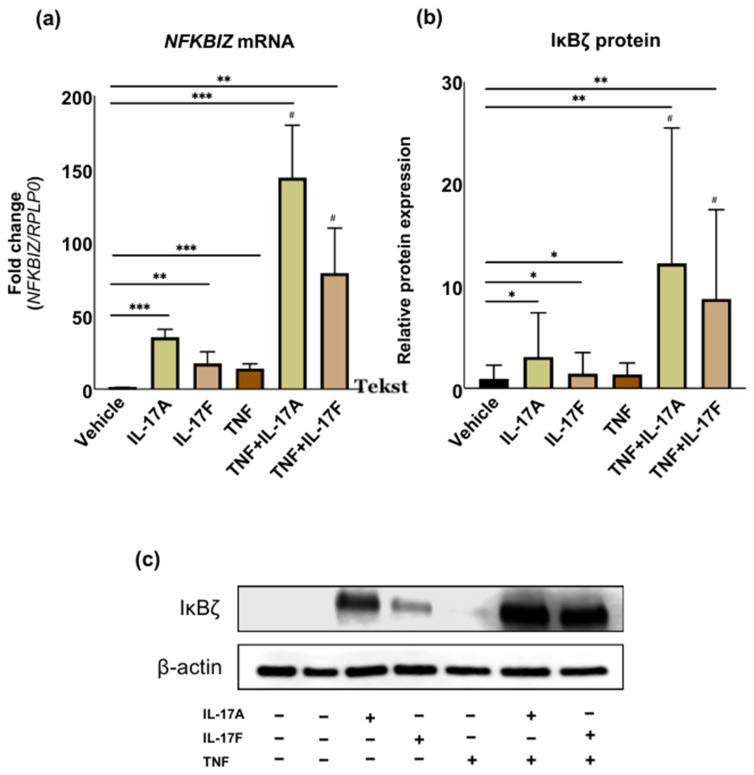
***NFKBIZ* and IκBζ are Induced by IL-17 Cytokines in Dermal Fibroblasts.** Cultured human fibroblasts were stimulated with IL-17A (100 ng/mL), IL-17F (100 ng/mL), TNF (10 ng/mL), and a combination of IL-17 cytokines with TNF for 2 h. (**a**) The expression of *NFKBIZ* mRNA was measured using qPCR (*n* = 5) and presented as mean ± standard deviation. *RPLP0* was utilized as a reference for normalization. (**b**,**c**) Protein levels of IκBζ were analyzed by Western blotting (*n* = 5). The quantification of the Western blot data displays the relative protein expression levels of IκBζ. β-actin was utilized as a loading control. Data are presented as mean ± SD, and statistical significance was assessed by Student’s *t*-test comparing each treatment group to the vehicle control. Synergism (#) was determined by comparing the observed combined effect to the calculated additive effect using Student’s *t*-test. * *p* < 0.05, ** *p* ≤ 0.01, *** *p* ≤ 0.001.

Numerous studies highlight *NFKBIZ’s* role in psoriasis susceptibility, yet its link to dermal fibroblasts remains unclear [1,3,4,17]. Primary human dermal fibroblasts were cultured in vitro and stimulated with psoriasis-associated cytokines for 2 h to investigate the induction of the *NFKBIZ* gene with qPCR (Figure 1a). Individually, stimulation with IL-17A, IL-17F, and TNF resulted in a modest impact on *NFKBIZ* mRNA expression after 2 h, with fold inductions of 35, 17.5, and 14, respectively. This contrasted with the combined stimulation, where IL-17A and TNF co-stimulation led to a substantial 143-fold increase in *NFKBIZ* mRNA expression, while IL-17F and TNF co-stimulation resulted in an approximately 79-fold increase. Furthermore, the combination of TNF and IL-17A or IL-17F stimulation resulted in a significant synergistic increase in gene expression that was higher than the additive values of the individual stimuli. IL-17A was the most potent inducer of *NFKBIZ* expression in dermal fibroblasts, both alone and in combination with TNF.

We then investigated whether the observed induction of *NFKBIZ* mRNA expression corresponded with increased protein levels of IκBζ (Figure 1b,c). The Western blot analysis confirmed increased IκBζ protein levels following cytokine stimulation. These findings demonstrate that IL-17 cytokines induce *NFKBIZ* and IκBζ expression in dermal fibroblasts, suggesting that IκBζ may also contribute to the transcriptional regulation of dermal cells in psoriatic skin.

### 2.2. NF-κB-Dependent Mechanism Mediates the Induction of NFKBIZ in Dermal Fibroblasts

**Figure 2:**
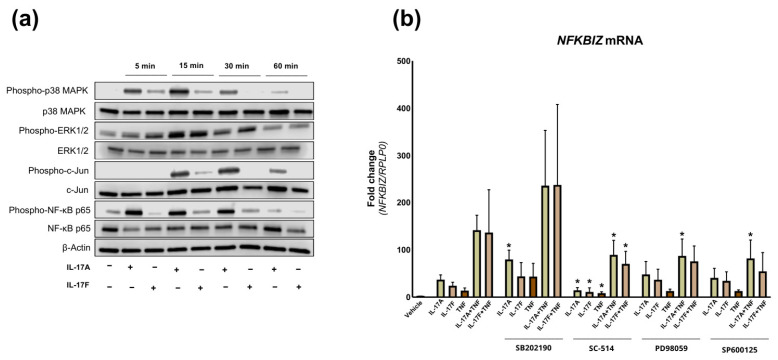
**Regulation of *NFKBIZ* mRNA expression in Fibroblasts by NF-κB Signaling.** (**a**) Cultured human fibroblasts were stimulated with IL-17A (100 ng/mL), IL-17F (100 ng/mL), TNF (10 ng/mL), and a combination of IL-17 cytokines with TNF for 5, 15, 30, and 60 min. The phosphorylation status of key signaling molecules was determined by Western blotting (*n* = 2). β-actin served as the loading control. (**b**) Cultured human fibroblasts were preincubated with either a p38 MAPK inhibitor (SB202190), NF-κB inhibitor (SC-514), JNK inhibitor (SP600125), or ERK1/2 inhibitor (PD98059) before stimulation with IL-17A, IL-17F, TNF, and a combination of IL-17 cytokines with TNF for 2 h (*n* = 5). The expression of *NFKBIZ* mRNA was measured using qPCR and presented as mean ± standard deviation. *RPLP0* served as the reference for normalization. Statistical significance was assessed by Student’s *t*-test, comparing each inhibitor-treated group to its respective cytokine-stimulated control without inhibitor. * *p* < 0.05.

To further characterize *NFKBIZ* induction in dermal fibroblasts, we investigated the molecular pathways related to its gene regulation. Stimulation of keratinocytes with TNF and IL-17 cytokines has been shown to activate both MAPK and NF-κB pathways [18,19,20]. To assess the activation of key signaling pathways in dermal fibroblasts, protein extracts from cultured fibroblasts stimulated with IL-17A or IL-17F for 5, 15, 30, and 60 min were analyzed by Western blotting, with phosphorylation status serving as an indicator of pathway activation (Figure 2a). Upon stimulation with IL-17A or IL-17F, phosphorylation occurred across all four key signaling molecules: p38 MAPK, NF-κB/p65, ERK1/2, and c-Jun. P38 MAPK, NF-κB/p65, and ERK1/2 phosphorylation occurred after 5 min of stimulation, whereas c-Jun phosphorylation was observed after 15 min. Collectively, the activation of the MAPK and NF-κB pathways in stimulated dermal fibroblasts was demonstrated by phosphorylation of key signaling molecules, mirroring the patterns observed in keratinocytes [18].

To investigate which of these signaling pathways were specifically involved in the induction of *NFKBIZ* in dermal fibroblasts, we utilized the following chemical inhibitors 45 min before stimulation: SB202190 (p38 MAPK inhibitor), PD98059 (ERK1/2 inhibitor), SP600125 (N-terminal kinase (JNK) inhibitor) and SC-514 inhibitor (NF-κB kinase 2 (IKK2) inhibitor) (Figure 2b). Preincubation with SC-514 significantly reduced *NFKBIZ* mRNA expression in dermal fibroblasts induced by IL-17A, IL-17F, TNF, and combinations of these cytokines. The downregulation of TNF-induced *NFKBIZ* mRNA expression was also observed by inhibiting the ERK1/2 and JNK1/2 pathways (Figure 2b). These results suggest that *NFKBIZ* mRNA induction in dermal fibroblasts is regulated by NF-κB signaling, with ERK1/2 and JNK1/2 pathways specifically involved in TNF-induced expression.

### 2.3. NFKBIZ Knockdown Attenuates Cytokine-Induced Expression of Inflammatory Mediators in Dermal Fibroblasts

**Figure 3:**
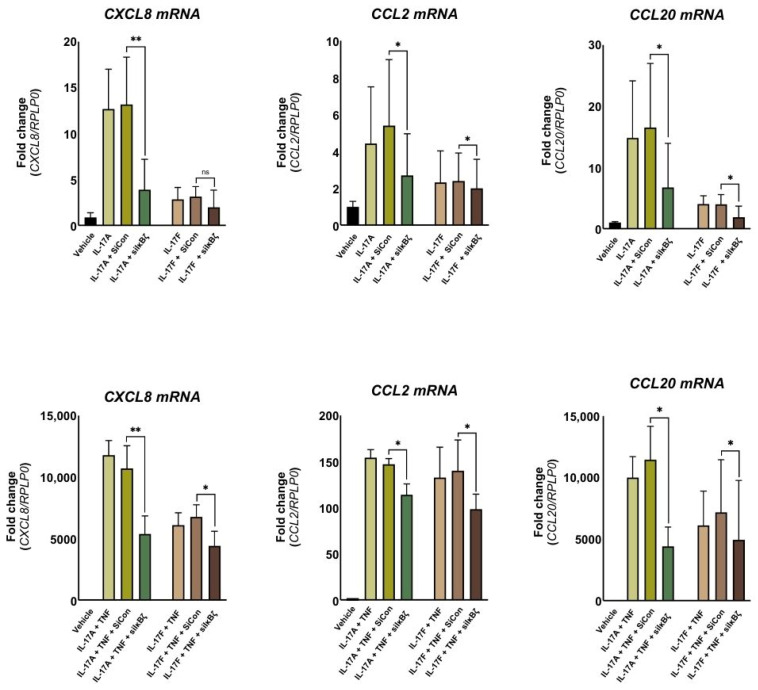
***NFKBIZ* knockdown reduces cytokine-induced expression of inflammatory mediators in dermal fibroblasts.** Primary human dermal fibroblasts were transfected with either control siRNA (siCon) or siRNA targeting IκBζ (siIκBζ) before stimulation with IL-17A (100 ng/mL), IL-17F (100 ng/mL), TNF (10 ng/mL), or their combinations for 24 h. *CXCL8*, *CCL2*, and *CCL20* gene expression levels were analyzed by qPCR (*n* = 4). Data are presented as mean ± standard deviation. *RPLP0* was used as a housekeeping gene. Statistical significance was assessed by Student’s *t*-test comparing each treatment group to the vehicle control. * *p* < 0.05, ** *p* ≤ 0.01.

Next, we explored whether the co-transcription factor IκBζ regulates gene expression of psoriasis-associated mediators in dermal fibroblasts [21,22,23]. To assess this, we measured *CXCL8*, *CCL2*, and *CCL20* mRNA expression using qPCR following a 24-h stimulation. To assess the role of IκBζ in the regulation of these cytokines, cultured dermal fibroblasts were transfected with IκBζ-specific small interfering RNA (siRNA) prior to stimulation. *NFKBIZ* silencing via siRNA resulted in a significant reduction in IκBζ protein expression by approximately 75–85% compared to cells transfected with control siRNA (Supplementary Figure S1). Stimulation with IL-17A, IL-17F, TNF or their combinations resulted in the upregulation of all selected inflammatory mediators compared with vehicle, with a synergistic effect observed when IL-17 cytokines were combined with TNF (Figure 3). *NFKBIZ* knockdown notably diminished the expression of IL-17A-induced inflammatory mediators, reducing *CXCL8*, *CCL20*, and *CCL2* mRNA expression by 50%, 63%, and 76%, respectively. In contrast, a lower induction and a smaller reduction in expression were observed for IL-17F-induced mRNA expression of *CCL2*, *CXCL8*, and *CCL20*. Under combined cytokine stimulation (IL-17A + TNF or IL-17F + TNF), *NFKBIZ* knockdown resulted in significant suppression of all three inflammatory mediators. Specifically, a reduction of approximately 50% was observed for *CXCL8* and *CCL20* mRNA expression under IL-17A + TNF stimulation, with a smaller decrease of 22% for *CCL2* mRNA expression. For IL-17F + TNF stimulation, all three mediators showed a roughly 30% reduction.

Collectively, these findings highlight IκBζ as a regulator of cytokine-induced inflammatory gene expression in dermal fibroblasts.

### 2.4. CosMx Spatial Molecular Imaging Confirms NFKBIZ Expression in Keratinocytes and Identifies Its Expression in Dermal Fibroblasts Within Psoriatic Lesions

**Figure 4:**
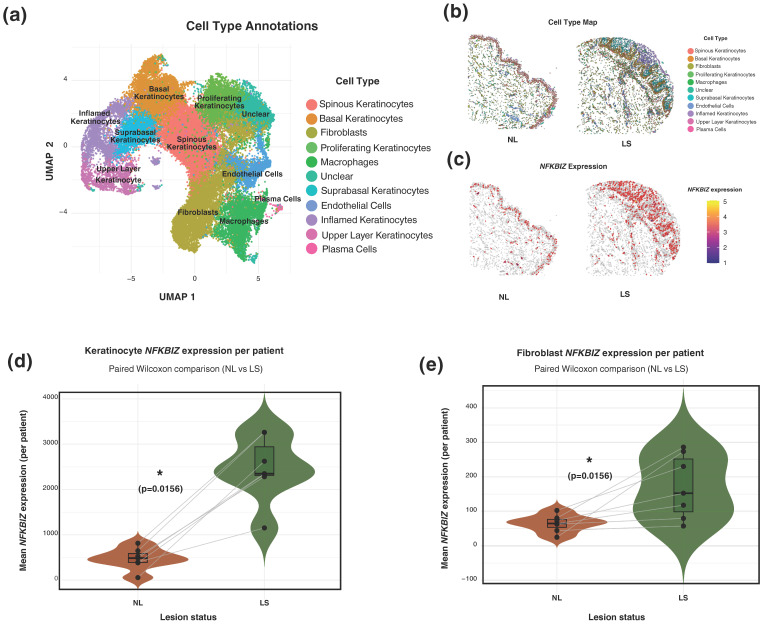
**Spatial Transcriptomics Confirms *NFKBIZ* Expression in Psoriatic Skin.** Paired lesional (LS) and non-lesional (NL) skin biopsies from patients with psoriasis vulgaris (*n* = 7) were analysed using the CosMx™ Spatial Molecular Imager (NanoString Technologies, Seattle, WA, USA). (**a**) UMAP embedding of all single cells identified across LS and NL samples showing unsupervised clustering and cell type annotations, including basal, suprabasal, upper-layer, spinouse, proliferating, and inflamed keratinocytes, fibroblasts, endothelial cells, macrophages, and plasma cells. (**b**) Spatial distribution of annotated cell types from a representative patient demonstrates marked architectural differences between NL and LS skin, including epidermal thickening and expansion of proliferating keratinocytes in LS tissue. (**c**) Spatial visualisation of *NFKBIZ* transcripts and cell-type distribution in the same fields of view. Cells expressing *NFKBIZ* (expression > 0) were visualised using filled polygons scaled by expression intensity, while *NFKBIZ*^−^ cells were outlined in light grey. For a representative patient with matched NL and LS samples, segmentation polygons were re-coloured by cell type. (**d**,**e**) Mean log-normalised *NFKBIZ* expression per patient in pooled keratinocytes (**d**) and fibroblasts (**e**), comparing NL and LS skin. Lines connect matched patient samples. Statistical significance for paired comparisons was assessed by the Wilcoxon signed-rank test. * *p* < 0.05.

To assess whether the induction of *NFKBIZ* observed in vitro in cytokine-stimulated dermal fibroblasts is present in vivo, we utilized single-cell level highly multiplexed transcriptomic analysis on paired non-lesional (NL) and lesional (LS) skin from patients with psoriasis (*n* = 7) using the CosMx™ Spatial Molecular Imager platform. UMAP clustering and supervised visual and gene-expression based analysis identified cell clusters corresponding to the main cell populations found in the skin, including basal and spinous keratinocytes, fibroblasts, endothelial cells, and various immune subsets (Figure 4a).

Spatial visualisation of the same tissue sections from one representative patient further demonstrated the localisation and distribution of cell types within the skin architecture (Figure 4b,c). In NL skin, keratinocytes formed a thin, well-defined epidermal layer overlying a structurally organised dermis composed primarily of fibroblasts and scattered immune cells (Figure 4b). In contrast, LS displayed pronounced epidermal thickening and a denser dermal cellular composition with increased numbers of fibroblasts and infiltrating immune cells (Figure 4b). Raw *NFKBIZ* expression was predominantly detected in the thickened psoriatic epidermis, but also in immune-rich dermal regions (Figure 4c).

Next, keratinocytes of all subtypes (suprabasal, basal, spinous, proliferating, inflamed and upper layer) were pooled and included as a reference cell type with known upregulation of *NFKBIZ* in psoriasis. Quantification of NFKBIZ mean expression at the pseudobulk single-cell level showed increased expression in both keratinocytes and fibroblasts in LS compared with matched NL samples (Figure 4d). LS dermal fibroblasts likewise displayed significantly elevated *NFKBIZ* expression. Feature plots projecting *NFKBIZ* expression onto the UMAP embedding confirmed its specific localization within the fibroblast cluster. Importantly, this visualization highlighted a marked increase in the density of *NFKBIZ*-positive fibroblasts in lesional compared to non-lesional skin (Supplementary Figure S2).

## 3. Discussion

Despite being the skin’s second most abundant cell type, dermal fibroblasts have been understudied in psoriasis research. While essential for extracellular matrix production and skin homeostasis, their role in inflammatory skin diseases has only recently gained attention. Our findings demonstrate that dermal fibroblasts actively participate in psoriasis pathogenesis through an IκBζ-dependent inflammatory response.

In resting cells, IκBζ is barely detectable, but under inflammatory conditions, it is highly upregulated in various cell types. Consistent with previous findings in keratinocytes, we observed significant upregulation of IκBζ expression in human dermal fibroblasts following stimulation with IL-17A and IL-17F [1]. While TNF stimulation elicited a minor effect on *NFKBIZ* mRNA expression alone, its co-stimulation with IL-17A or IL-17F synergistically induced *NFKBIZ* mRNA expression. The observed upregulation, consistent with prior investigations, exhibited greater potency upon stimulation with IL-17A than IL-17F, despite their considerable structural similarity [1]. This phenomenon has been attributed to IL-17A’s heightened affinity for the IL-17RA/RC receptor complex, a characteristic that may similarly manifest in dermal fibroblasts [24]. The mechanism behind the synergistic effect remains unclear, but IL-17 cytokines may stabilize mRNA and activate shared pathways with TNF, enhancing gene expression [25]. However, investigations within dermatological and rheumatological research highlight that this synergy is highly cell-type specific and involves diverse regulatory mechanisms depending on the specific gene [26,27,28]. In line with this, our research suggests IL-17 cytokines also directly activate *NFKBIZ* transcription, proposing a role in transcriptional regulation in addition to mRNA stabilization.

This study shows that the induction of *NFKBIZ* mRNA expression by IL-17 cytokines and/or TNF is regulated through NF-κB signaling pathways, with the ERK1/2 and JNK1/2 pathways specifically involved in TNF-induced *NFKBIZ* mRNA expression. These findings are close to our previous observations in keratinocytes [29,30]. Interestingly, inhibition of the p38 MAPK pathway resulted in an upregulation of *NFKBIZ* mRNA levels for all conditions, but only significantly during IL-17A stimulation alone. The pathway appears to negatively regulate *NFKBIZ* induction by IL-17 cytokines and TNF, in contrast to keratinocytes, where p38 promotes *NFKBIZ* expression, suggesting cell type-specific regulatory differences [18,29,30].

Given the critical role of IκBζ in regulating the transcription of several cytokines and antimicrobial peptides in keratinocytes, we aimed to explore its role in regulating mediators with chemotactic functions in psoriasis. CXCL8, CCL20, and CCL2 are all inflammatory mediators, described respectively to attract neutrophils, Th17 cells, and monocytes to sites of inflammation, thereby sustaining the chronic inflammatory environment characteristic of the disease [31,32,33]. Considering their strategic position in the dermal compartment, fibroblasts may act as amplifiers of IL-17-mediated inflammation by coordinating chemokine-driven immune cell recruitment. In our study, we identified IκBζ as a regulator of psoriasis-associated mediators in response to IL-17A and -F cytokines and their combination with TNF. These findings suggest that IκBζ serves as a transcriptional regulator of IL-17-driven responses in dermal fibroblasts, consistent with its established role in keratinocytes [1].

To confirm that these mechanisms are also relevant in vivo, we performed a single-cell spatial transcriptomic analysis. This analysis validated *NFKBIZ* expression in keratinocytes and, importantly, identified its expression in dermal fibroblasts within lesional psoriatic skin. Compared with non-lesional samples, fibroblasts exhibited significantly elevated *NFKBIZ* expression in lesional skin. Spatial mapping further demonstrated the characteristic architecture of psoriatic lesions, with epidermal hyperplasia, expansion of proliferating keratinocytes, and increased dermal cellularity. *NFKBIZ* transcripts were primarily localised to the epidermis but were also detected in dermal fibroblast-rich regions. These data support that dermal fibroblasts in psoriatic skin express *NFKBIZ* in vivo, strengthening the translational link between our in vitro findings and the disease context.

Although our study aligns with numerous investigations on IκBζ in psoriasis, a significant strength, several limitations should be considered. The CosMx™ SMI platform provides high-resolution spatial RNA data but measures transcript levels rather than protein expression, limiting conclusions about functional IκBζ activity in vivo. In addition, the analysis was based on 1.5-mm tissue microarray cores derived from 4-mm biopsies and included paired samples from only seven donors. While this limited sampling area and varying anatomical sites could introduce spatial heterogeneity, the consistent upregulation observed across paired samples supports the robustness of the findings, though a larger cohort would have strengthened the study. Spatial transcriptomics is inherently descriptive, underscoring the need for mechanistic studies in vivo using fibroblast-specific models to define the functional role. Moreover, our in vitro fibroblast experiments were conducted in standard culture with fetal bovine serum, where passage and matrix effects may alter phenotype. Finally, only a selected panel of IκBζ-regulated chemokines was investigated, limiting insight into the broader transcriptional program.

Despite these methodological constraints, the consistency between our in vitro and in vivo findings underscores the relevance of dermal IκBζ in psoriatic inflammation. Our findings support the therapeutic potential of IκBζ as a topical treatment in psoriasis. This localized approach circumvents the lethality observed in systemic knockout models [1] while effectively targeting the skin. Importantly, by demonstrating the role of IκBζ in dermal fibroblasts, we expand our understanding of disease pathogenesis, providing mechanistic insight into how IκBζ acts as a critical transcriptional co-activator to selectively amplify specific NF-κB target genes, driving chronic inflammation.

## 4. Materials and Methods

### 4.1. Cell Cultures

Healthy adult dermal fibroblasts were isolated from skin samples following surgery (*n* = 5) using the explant technique as previously described [34]. Briefly, skin biopsies were minced into small fragments and left undisturbed in culture flasks for 7–14 days to allow for cell migration and proliferation. Cells were cultured in Dulbecco’s Modified Eagle Medium (DMEM) (Gibco, ThermoFisher Scientific, Waltham, MA, USA), supplemented with 10% fetal bovine serum (FBS) (Gibco, ThermoFisher Scientific, Waltham, MA, USA) and antibiotics (penicillin/streptomycin (10 μg/mL), gentamicin (5 μg/mL)). The number of biological replicates (*n*) for each experiment is specified in the figure legends. The cells were cultured at 37 °C in a humidified atmosphere containing 5% CO_2_ until 70–80% confluency. The medium was replaced with a fibroblast basal medium (DMEM with 2% FBS) before stimulation. Cells were treated with either vehicle (PBS containing 0.15% bovine serum albumin (BSA)), IL-17A (100 ng/mL), IL-17F (100 ng/mL), or TNF (10 ng/mL) (PeproTech, ThermoFisher Scientific, Waltham, MA, USA), individually or in combinations consisting of IL-17A + TNF and IL-17F + TNF. To delineate the signaling pathways mediating *NFKBIZ* induction, dermal fibroblasts were pretreated for 45 min with selective inhibitors targeting different signaling pathways: SB202190 (p38 MAPK, 10 µM), SC-514 (NF-κB, 50 µM), PD98059 (ERK1/2, 50 µM), or SP600125 (JNK1/2, 20 µM) (Merk Millipore, Burlington, MA, USA). Cell collection occurred at various time intervals depending on the specific experimental protocol.

### 4.2. siRNA Transfection

Human dermal fibroblasts were cultured until they reached approximately 75% confluency. Immediately before transfection, the medium was replaced with fresh growth medium (DMEM supplemented with 10% FBS). For targeted silencing of *NFKBIZ*, ON-TARGETplus SMARTpool siRNA (Dharmacon RNA Technologies, Lafayette, CO, USA) specific to IκBζ was used. The siRNA was mixed with Lipofectamine 2000 (Invitrogen, Thermo Fisher Scientific, MA, USA) in a serum-free medium to form siRNA-lipoplex complexes, which were incubated for 10 min at room temperature. The siRNA-lipoplex complexes were then introduced to the fibroblast cultures, achieving a final siRNA concentration of 150 nM. Cells were transfected with a non-targeting siRNA pool (Dharmacon RNA Technologies) under identical conditions as a negative control. Following a 24-h incubation period post-transfection, the medium was replaced with basal medium (DMEM containing 2% FBS) to prepare the cells for subsequent stimulation experiments.

### 4.3. RNA Isolation

Dermal fibroblasts were rinsed twice with ice-cold phosphate-buffered saline (PBS; Gibco, ThermoFisher Scientific, Waltham, MA, USA) to remove residual media. RNA was isolated using the SV 96 Total RNA Isolation System (Promega, Madison, WI, USA) following the manufacturer’s protocol. RNA concentration and purity were assessed using a NanoDrop™ 2000 spectrophotometer (ThermoFisher Scientific, Waltham, MA, USA).

### 4.4. Quantitative Polymerase Chain Reaction

TaqMan™ Reverse Transcription Reagents (Gibco, ThermoFisher Scientific, Waltham, MA, USA) and a PTC-200 Peltier Thermal Cycler (MJ Research Inc, Waltham, MA, USA) were utilized for cDNA generation. Real-time PCR was carried out employing Platinum^®^ qPCR Supermix-UDG (Gibco, Life Technologies, Austin, TX, USA) with TaqMan™ primers and probes on a Rotor-Gene Q Real-Time PCR cycler (Corbett Research, Sydney, Australia), following manufacturer instructions. The following primer and probes were used: human *NFKBIZ* (assay ID: Hs00230071_m1), *CXCL8* (assay ID: Hs00174103_m1), *CCL20* (assay ID: Hs00355476_m1), and *CCL2* (assay ID: Hs00234140_m1). Each gene was analyzed in triplicate, and a standard curve was established based on a 4-fold dilution of total RNA. *RPLP0* (assay ID: Hs99999902_m1) was used as a housekeeping gene. Data analysis was performed using the RotorGene software v2.3.103.23 (Qiagen, Hilden, Germany), Microsoft Excel (Microsoft, Redmond, WA, USA), and GraphPad Prism 10 (GraphPad Software, San Diego, CA, USA).

### 4.5. Protein Isolation

Cells were washed with PBS and lysed in buffer supplemented with phenylmethylsulfonyl fluoride and Complete Protease Inhibitor Cocktail (Sigma-Aldrich, St. Louis, MO, USA). After centrifugation at 13,000× *g* for 3 min, the supernatant was collected. Protein concentration was determined using the Bio-Rad Protein Assay and measured on a Thermo Scientific Multiskan GO (ThermoFisher Scientific, Waltham, MA, USA).

### 4.6. Western Blotting

Equal protein amounts were separated by SDS-PAGE and blotted onto nitrocellulose membranes. Membranes were incubated with the following antibodies: anti-IκBζ (#9244), anti-p-p38 MAPK (#9211), anti-p38 MAPK (#9212), anti-p-p44/42 MAPK (#9101), anti-p44/42 MAPK (#9102), anti-p-c-Jun (#9261), anti-c-Jun (#9165), anti-p-NF-κB p65 (#3033), anti-NF-κB p65 (#8242) and anti-β-actin (#8457) (Cell Signaling Technology, Danvers, MA, USA). The antibodies were detected using an anti-rabbit IgG-HRP conjugate (Cell Signaling Technology, Danvers, MA, USA) or an anti-mouse IgG-HRP conjugate (Dako, Glostrup, Denmark). Detection was performed using a standard enhanced chemiluminescence (ECL) reaction (Amersham Biosciences, Piscataway, NJ, USA) according to the manufacturer’s guidelines.

### 4.7. Spatial Transcriptomics

Paired lesional (LS) and non-lesional (NL) four-mm skin punch biopsies were obtained from patients with psoriasis vulgaris (*n* = 7) after written informed consent (permission number: 1-10-72-10-24, The Central Denmark Region Committees on Health Research Ethics). Detailed patient demographics are provided in Supplementary Table S1. Samples were fixed in neutral buffered formalin and embedded in paraffin (FFPE). Tissue microarrays (TMAs) were subsequently constructed from each paraffin-embedded biopsy by extracting 1.5-mm cores and assembling them into recipient blocks. Sections from the TMA blocks were cut at 5 µm thickness, placed on TOMO slides for use in the CosMx™ Spatial Molecular Imager (NanoString Technologies), and processed at the Department of Molecular Medicine (MOMA) at Aarhus University Hospital as described in the Nanostring Manual (CosMx SMI Instrument User Manual [35]) and previous literature [36].

Spatial transcriptomic profiling was performed using the CosMx™ Human 6000-plex RNA Panel following the manufacturer’s protocol. FFPE sections were deparaffinized, subjected to antigen retrieval and permeabilization, and treated with Proteinase K (2 µg/mL). Sections were then hybridized with fluorescently barcoded oligonucleotide probes targeting predefined transcripts. Morphology markers, including DNA, PanCK, CD45, CD68 and a general membrane stain, were applied to support nuclear and cell segmentation. The CosMx SMI instrument subsequently executed iterative cycles of hybridisation, imaging, fluorophore cleavage and probe stripping, enabling transcript decoding based on unique colour barcodes and the generation of spatially resolved, single-cell expression data for downstream analysis.

Image processing, transcript decoding, and segmentation were performed using CosMx™ SMI software, CosMx Data Analysis (v2.1) (AtomX). The resulting cell-by-gene count matrix was exported for downstream analysis in R (v4.3.2). Fields of view (FOVs) were visually inspected to ensure consistent results. A preprocessed Seurat object containing CosMx-derived single-cell gene expression data was imported. Normalization and variance stabilization were performed on the raw count matrix (RNA assay, counts layer) using SCTransform [37]. Dimensionality reduction and clustering were performed using the Seurat R package (v5.3.0). Highly variable genes were identified, and principal component analysis (PCA) was applied to the scaled expression data (30 components). Nearest-neighbour graphs were constructed, and clusters were identified using the Louvain algorithm (resolution = 0.6) (FindNeighbors and FindClusters functions). Two-dimensional Uniform Manifold Approximation and Projection (UMAP) embeddings were generated for data visualisation. Cell clusters were annotated based on canonical marker gene expression derived from FindAllMarkers (minimum expression in ≥25% of cells per cluster, log_2_ fold-change > 0.25) (Supplementary Table S2), aided by putative spatial location. A complete list of R packages, versions, and references is provided in Supplementary Table S3. For cell type-specific analyses, cells annotated as fibroblasts or keratinocytes were extracted as pooled clusters from the dataset. For each patient and lesion status (NL and LS), the mean LogNormalized raw RNA expression of *NFKBIZ* was calculated using the AggregateExpression function. Paired comparisons between NL and LS samples were performed using a Wilcoxon signed-rank test in R. Statistical significance was defined as a two-sided *p* < 0.05. Spatial plots were generated from per-cell x-y coordinates and cell-type annotations using the ggplot2 package (v4.0.0). To visualize the spatial distribution of *NFKBIZ* transcripts, polygon segmentation boundaries were extracted.

### 4.8. Statistical Analysis

All analyses and graphs were produced with GraphPad Prism version 10.6.0 or RStudio version 4.3.2. Data are presented as individual values and mean ± standard deviation (SD) with statistical significance assessed by Student’s *t*-test or Wilcoxon signed-rank as appropriate. The symbol “#” is utilized to denote synergistic effects. Synergism was defined as a significantly greater response observed with combined stimulation of TNF and IL-17A, as well as TNF and IL-17F, compared to the additive effect of the cytokines alone. A significance level of *p* ≤ 0.05 was considered statistically significant and is denoted by *, while ** and *** represent *p*-values corresponding to *p* ≤ 0.01 and *p* ≤ 0.001, respectively.

## Data Availability

The spatial transcriptomics data presented in this study are openly available in the Gene Expression Omnibus (GEO) [GSE314158]. All other data are available on request from the corresponding author.

## Supplementary Materials

The supporting information can be downloaded at: https://www.mdpi.com/article/10.3390/ijms27031297/s1.

## Abbreviations

IκBζ	Inhibitor of NF-κB zeta
IL	Interleukin
TNF	Tumor Necrosis Factor
MAPK	Mitogen-Activated Protein Kinase
NF-κB	Nuclear Factor kappa B
scRNA-seq	Single-cell RNA sequencing
DMEM	Dulbecco’s Modified Eagle Medium
FBS	Fetal Bovine Serum
PBS	Phosphate-Buffered Saline
BSA	Bovine Serum Albumin
JNK	c-Jun N-terminal Kinase
ERK	Extracellular Signal-Regulated Kinase
siRNA	Small Interfering RNA
qPCR	Quantitative Polymerase Chain Reaction
HRP	Horseradish Peroxidase
ECL	Enhanced Chemiluminescence
LS	Lesional Skin
NL	Non-Lesional Skin
FFPE	Formalin-Fixed Paraffin-Embedded
TMA	Tissue Microarray
SMI	Spatial Molecular Imager
PCA	Principal Component Analysis
UMAP	Uniform Manifold Approximation and Projection
SD	Standard Deviation
