# Supplementary figures and images for: IL-17 Cytokines Induce IκBζ in Dermal Fibroblasts to Promote Pro-Inflammatory Gene Expression in Psoriasis
Source: Int J Mol Sci. 2026 Jan 28;27(3):1297. doi: 10.3390/ijms27031297 (PMC12898262; doi:10.3390/ijms27031297)

**(a)**

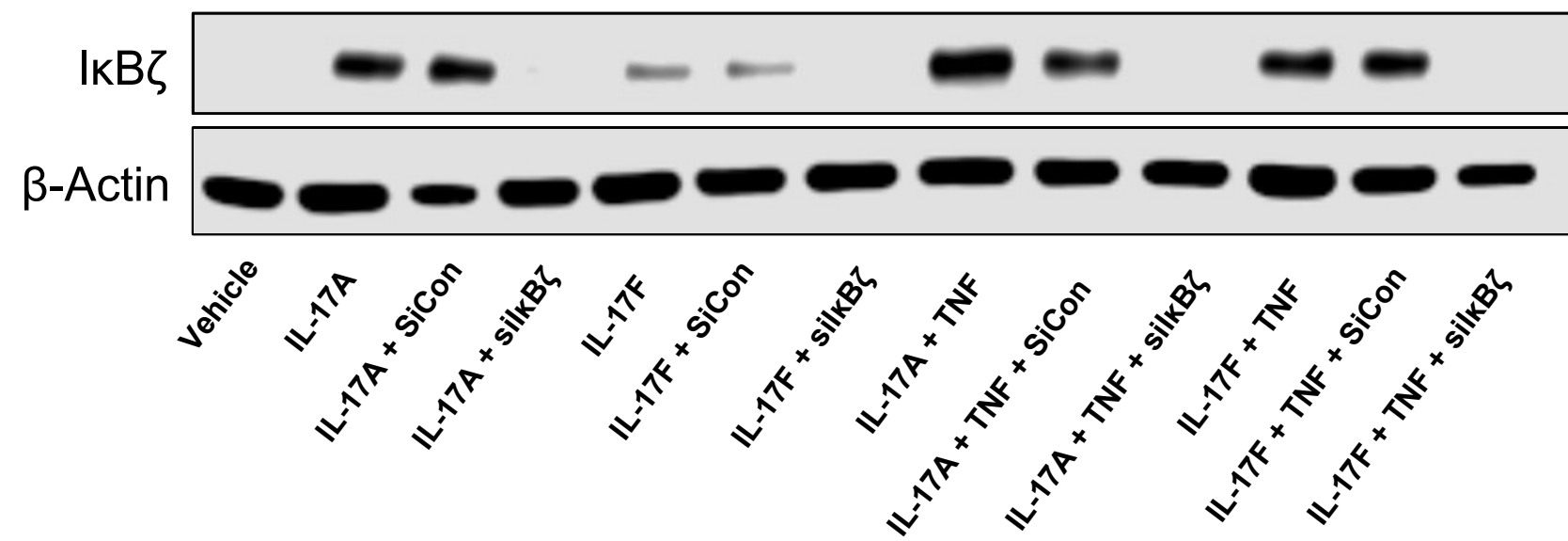

**(b)**

*IκBζ protein*

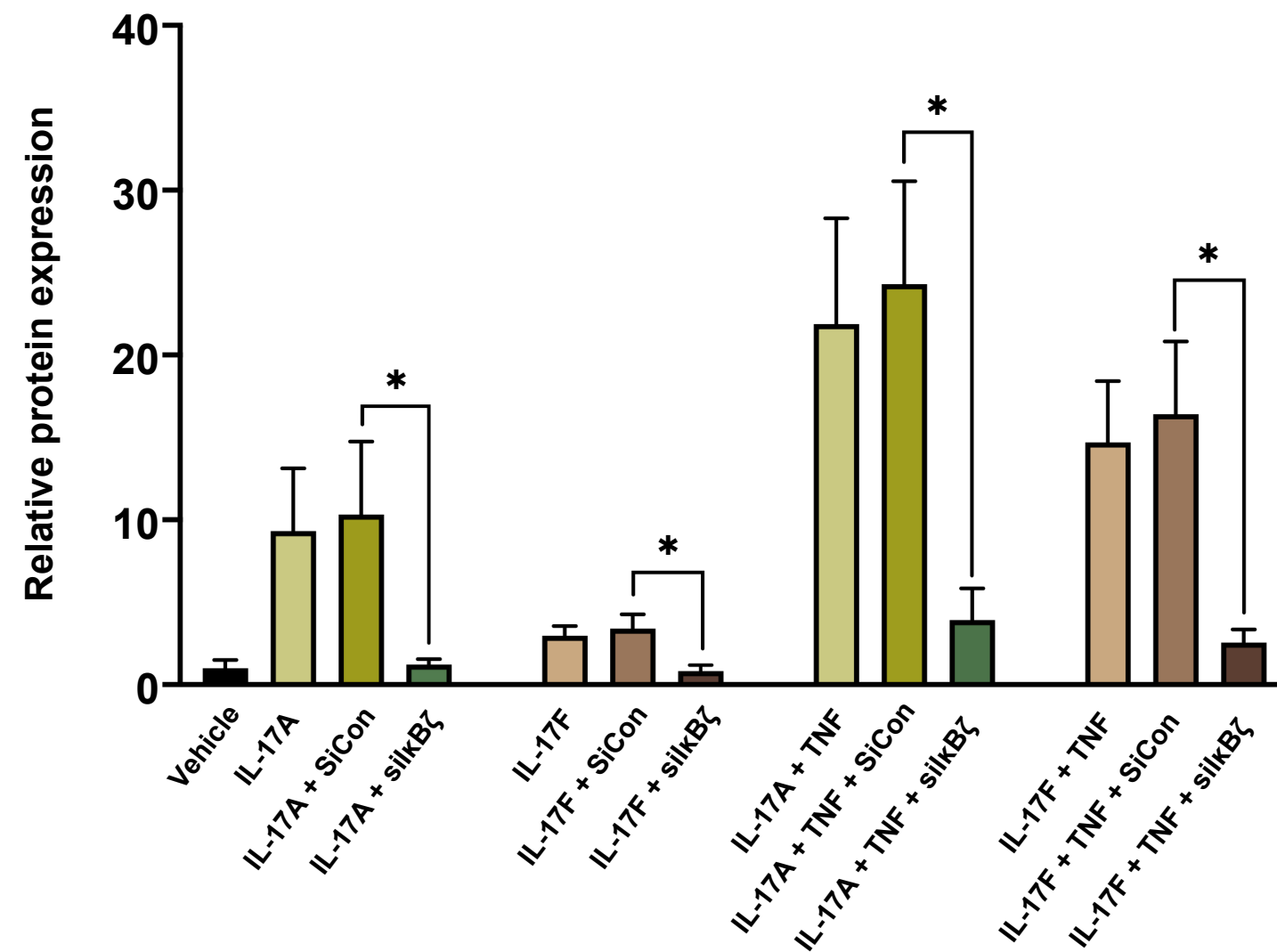

Supplement: Supplementary file 1 [file ijms-27-01297-s001.zip › Supplementary Figure S1-Validation of siRNA-mediated NFKBIZ knockdown efficiency in human dermal fibroblasts.pdf]

LS

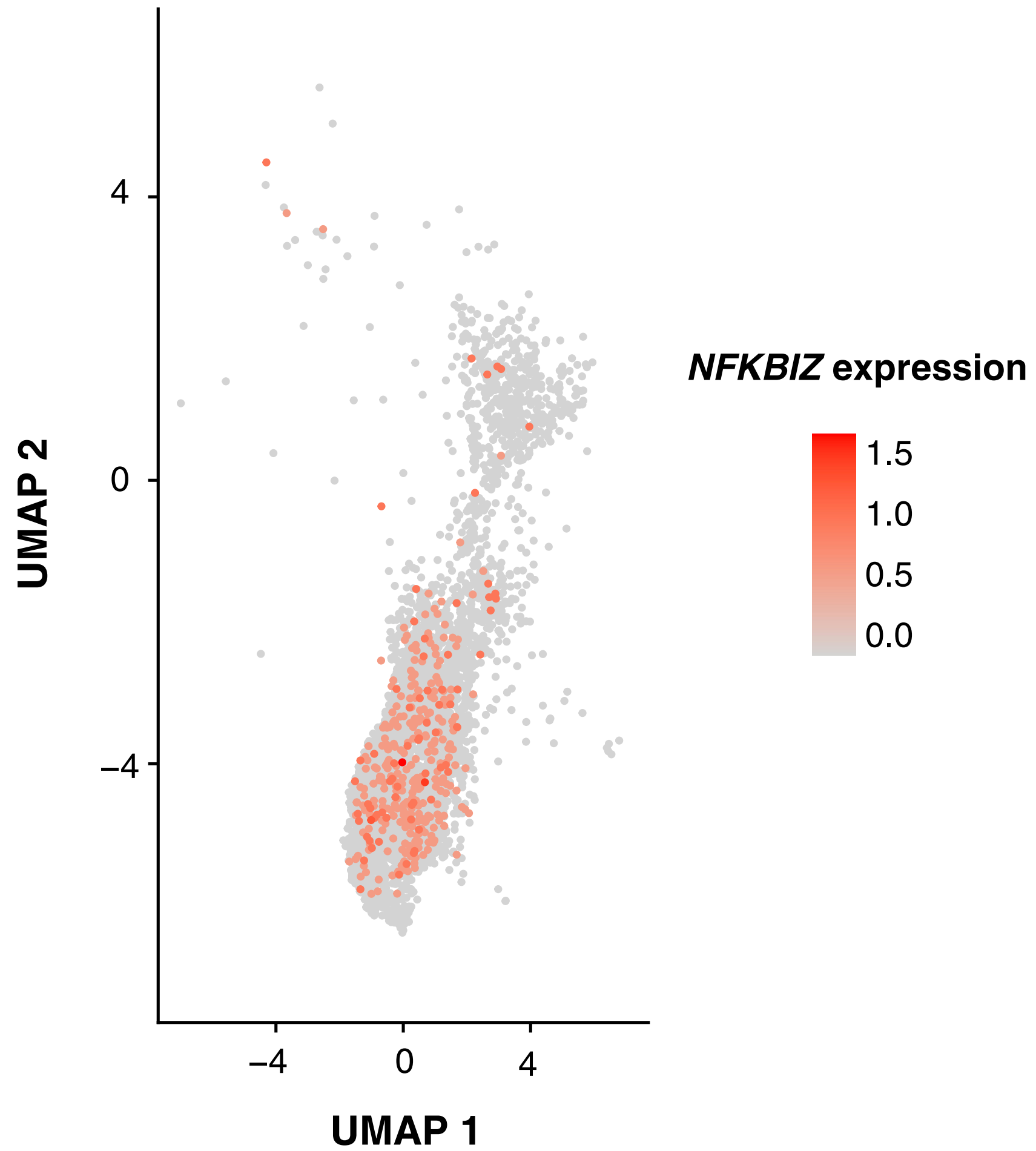

NL

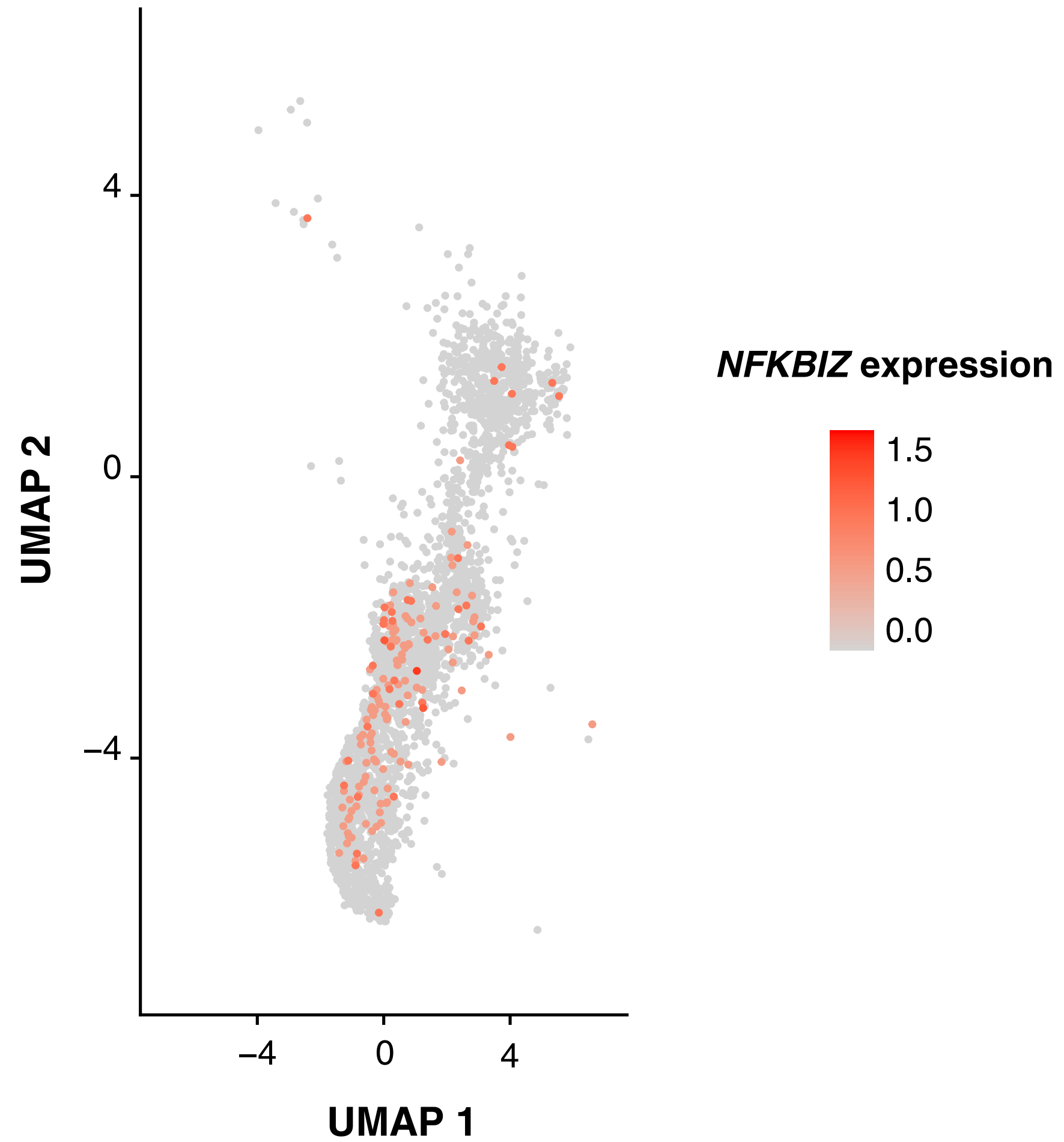

Supplement: Supplementary file 1 [file ijms-27-01297-s001.zip › Supplementary Figure S2-Feature Plots of NFKBIZ Expression in Lesional and Non-Lesional Psoriatic Skin.pdf]
